# Cilostazol Activates Function of Bone Marrow-Derived Endothelial Progenitor Cell for Re-endothelialization in a Carotid Balloon Injury Model

**DOI:** 10.1371/journal.pone.0024646

**Published:** 2011-09-12

**Authors:** Rie Kawabe-Yako, Ii Masaaki, Osamu Masuo, Takayuki Asahara, Toru Itakura

**Affiliations:** 1 Group of Vascular Regeneration Research, Institute of Biomedical Research and Innovation, RIKEN Center for Developmental Biology, Kobe, Japan; 2 Department of Neurosurgery, Wakayama Medical University, Wakayama, Japan; 3 Group of Translational Stem Cell Research, Department of Pharmacology, Osaka Medical College, Osaka, Japan; 4 Department of Regenerative Medicine Science, Tokai University School of Medicine Kanagawa, Japan; University of Padova, Italy

## Abstract

**Background:**

Cilostazol(CLZ) has been used as a vasodilating anti-platelet drug clinically and demonstrated to inhibit proliferation of smooth muscle cells and effect on endothelial cells. However, the effect of CLZ on re-endothelialization including bone marrow (BM)-derived endothelial progenitor cell (EPC) contribution is unclear. We have investigated the hypothesis that CLZ might accelerate re-endothelialization with EPCs.

**Methodology/Principal Findings:**

Balloon carotid denudation was performed in male Sprague-Dawley rats. CLZ group was given CLZ mixed feed from 2weeks before carotid injury. Control group was fed normal diet. CLZ accelerated re-endothelialization at 2 weeks after surgery and resulted in a significant reduction of neointima formation 4 weeks after surgery compared with that in control group. CLZ also increased the number of circulating EPCs throughout the time course. We examined the contribution of BM-derived EPCs to re-endothelialization by BM transplantation from Tie2/lacZ mice to nude rats. The number of Tie2-regulated X-gal positive cells on injured arterial luminal surface was increased at 2 weeks after surgery in CLZ group compared with that in control group. In vitro, CLZ enhanced proliferation, adhesion and migration activity, and differentiation with mRNA upregulation of adhesion molecule integrin αvβ3, chemokine receptor CXCR4 and growth factor VEGF assessed by real-time RT-PCR in rat BM-derived cultured EPCs. In addition, CLZ markedly increased the expression of SDF-1α that is a ligand of CXCR4 receptor in EPCs, in the media following vascular injury.

**Conclusions/Significance:**

CLZ promotes EPC mobilization from BM and EPC recruitment to sites of arterial injury, and thereby inhibited neointima formation with acceleration of re-endothelialization with EPCs as well as pre-existing endothelial cells in a rat carotid balloon injury model. CLZ could be not only an anti-platelet agent but also a promising tool for endothelial regeneration, which is a key event for preventing atherosclerosis or restenosis after vascular intervention.

## Introduction

Re-endothelialization inhibits neointimal thickening, thereby suppressing development of the substrate for lipid deposition and macrophage accumulation that is believed to induce the formation of atherosclerotic lesions and may contribute to restenosis. Drug-eluting stents (DESs) have significantly reduced the rate of restenosis; however, DESs also appear to delay re-endothelialization [1]. This delay results in excessive rates of thrombosis, which could increase the occurrence of acute coronary syndromes. Endothelial cell loss from arterial wall resulting from mechanical removal (hemodynamic forces, PTCA, stenting) or cell apoptosis, might induce a cascade of events giving rise to vascular inflammation, smooth muscle cells proliferation and activation of thrombosis and lead to neointimal hyperplasia and vascular remodeling, eventually inducing restenosis, that is key features of atherosclerosis development, progression and complication. Thrombosis occurs as a consequence of the exposure of thrombogenic surfaces, both stent and denuded vascular wall, to blood stream. Therefore, acceleration of re-endothelialization is a very useful not only to repair endogenously injured vessels, but also to reduce neointimal formation and prevent intrastent restenosis and atherosclerosis development.

Endogenous re-endothelialization is driven not only by migration and proliferation of resident endothelial cells (ECs) adjacent to sites of injury, but also with the activity of endothelial progenitor cells (EPCs). Studies performed in our laboratory and others demonstrated that both exogenously infused EPCs and EPCs derived from bone marrow (BM) which can be mobilized to circulation by ischemia [2], [3], physical training [4], and the administration of statins [5], [6], estrogen [7], [8], and a variety of cytokines [9], [10], [11], recruited to sites of arterial injury, where they promote re-endothelialization directly by the differentiation into mature endothelial cells and also indirectly by stimulating resident ECs and enhancing above process via EPC-released cytokines.

Cilostazol (CLZ) is a selective inhibitor of phosphodiesterase 3 (PDE3), and CLZ increases intracellular cAMP content and activates protein kinase A (PKA) [12], resulting in antiplatelet aggregation and peripheral vasodilatation. CLZ has therefore been used as a vasodilating anti-platelet drug for the treatment of ischemic symptoms in chronic peripheral arterial obstruction or intermittent claudication and for preventing recurrence of cerebral infarction [13], [14]. CLZ also inhibits vascular smooth muscle cell proliferation and has been shown to reduce neointima formation following arterial injury in animal models [15], [16], [17]. It has also been demonstrated that CLZ reduces post-procedural in-stent restenosis (ISR) after coronary artery stenting in the CREST trial [18], [19] and carotid artery stenting. [20] For the mechanistic insight of anti-neointimal formation, CLZ was shown to protect ECs from apoptosis induced by serum deprivation, high d-glucose, and lipopolysaccharide (LPS) [21], [22] via a hepatocyte growth factor production [23] and a suppression of superoxide production induced by remnant lipoprotein particles [24]. Moreover, it was reported that CLZ attenuated the expression of vascular cell adhesion molecule-1 (VCAM-1) [25] and monocyte chemoattractant protein-1 (MCP-1) [26] and intercellular adhesion molecule (ICAM-1) and P-selectin [27], as a result, CLZ prevented monocyte or neutrophil adhesion to endothelial cells. CLZ has pleiotropic effects on vascular remodeling following injury as described above, however, the effect of CLZ on re-endothelialization, specifically, including EPC contribution has not been investigated. We therefore tested the hypothesis that CLZ might accelerate re-endothelialization in a rat carotid balloon injury model, and analyzed the pathophysiological functions of CLZ in EPC biology.

## Results

### Cilostazol Accelerates Re-endothelialization in Injured Artery

**Figure 1:**
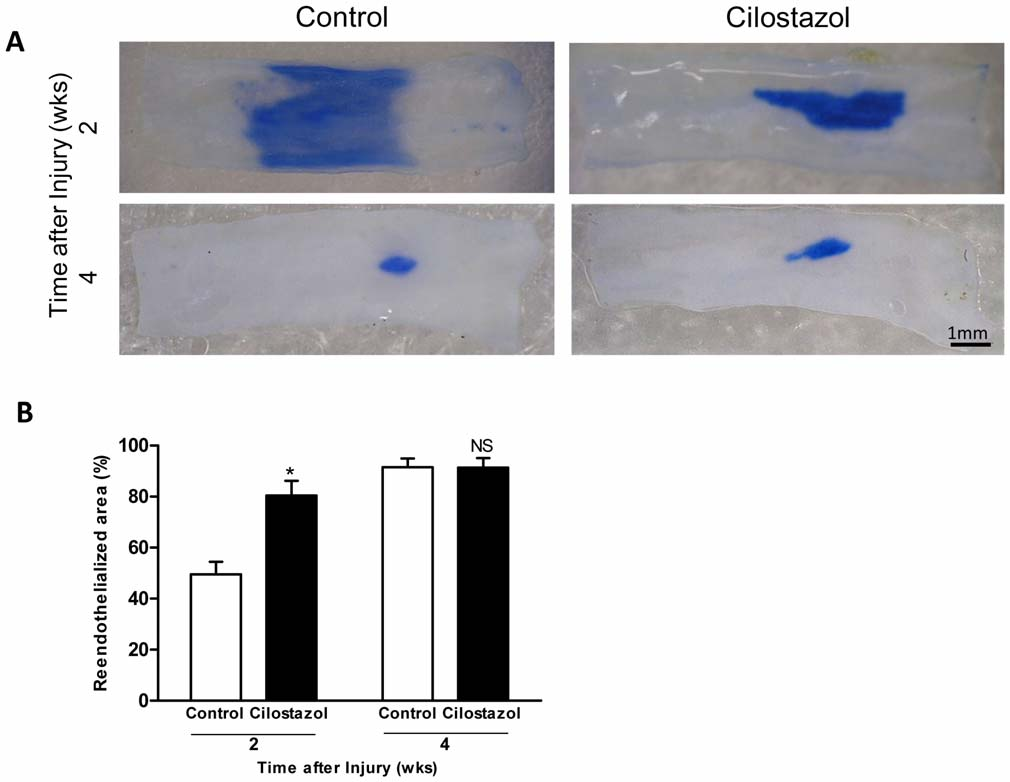
Cilostazol accelerates reendothelialization in injured carotid artery. (A) Rats were given cilostazol mixed feed (Cilostazol group) or normal diet (Control group) 2 weeks prior to carotid injury until time of sacrifice. Representative photomacrographs of Evans Blue dye staining of whole-mount en-face carotid arteries at 2 and 4 weeks after carotid balloon denudation. White area represent injured arterial wall covered with regenerated endothelium, and blue area represent injured arterial wall without endothelium. (B) Quantification of reendothelialized area was expressed as mean±SEM. *, *P<*0.001 and NS vs. Control (n = 7 in each group).

To evaluate the effect of CLZ on re-endothelialization, the carotid endothelial recovery following balloon denudation was assessed by Evans Blue staining. CLZ treatment accelerated re-endothelialization in the balloon-injured arterial segments. (Figure 1A) At 2 weeks, the re-endothelialized area in the CLZ-treated rats (n = 7) was 80.4±5.8% of the total denuded area. In contrast, the re-endotheliialized area was limited to 49.6±4.9% of the total denuded area in the control group (n = 7) (*P<*0.001). At 4 weeks, re-endothelialized area in both control group and CLZ group were around 90% of the denuded area (91.6±3.4% vs. 91.4±3.7%, NS), suggesting that although re-endothelialization was almost completed at 4 weeks in both groups CLZ accelerated re-endothelialization in subacute phase which is critical timing for preventing neointimal development following arterial injury. (Figure 1B).

### Cilostazol Inhibits Neointima Formation following Arterial Injury

**Figure 2:**
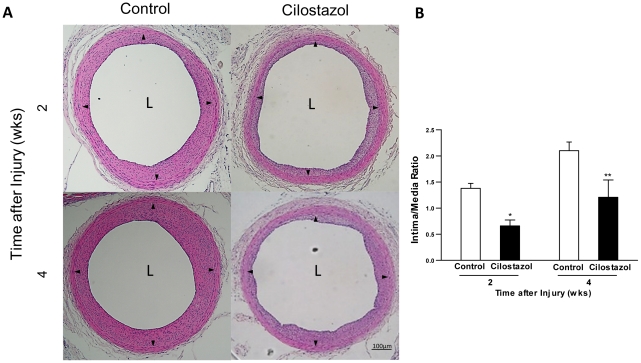
Cilostazol reduces neointima formation in injured carotid artery. (A) Rats were fed with Cilostazol containing diet (Cilostazol group) or normal diet (control group) from 2 weeks prior to carotid injury until time of sacrifice. Representative photomicrographs of H.E. stained histological cross sections in Cilostazol group (n = 5) vs. Control group (n = 6) at 2 and 4weeks after carotid injury. Black arrows indicate internal elastic lamina. (B) Intima/Media ratio was expressed as mean±SEM. *, *P<*0.05 and **P<0.01 vs. control.

The effect of CLZ on neointimal thickening was also examined at 2 and 4 weeks after carotid injury. (Figure 2A) In control rats (n = 13), intimal area/medial area (I/M) ratios increased markedly at 2 weeks (1.38±0.10) and 4 weeks (2.10±0.17). In contrast, I/M ratios of animals treated with CLZ (n = 13) were 0.66±0.11 at 2weeks and 1.21±0.33 at 4weeks (*P<*0.05 vs. control animals). (Figure 2B) CLZ exhibited statistically significant reduction of neointimal thickening as well as acceleration of re-endothelialization compared with controls.

### Cilostazol Increases Number of Circulating EPCs

**Figure 3:**
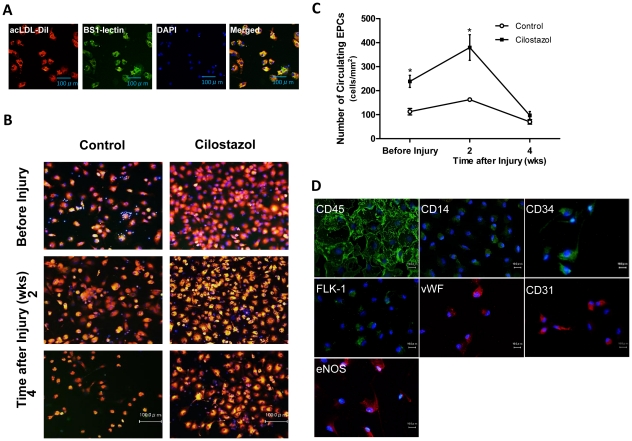
Representative fluorescent images of rat EPC culture assay. (A) Mononuclear cells isolated from 500 µl of peripheral blood were cultured in EPC differentiation medium for 4days, and stained with acLDL/DiI (red), FITC-conjugated BS-1 lectin (green) and DAPI (blue). (B) Cilostazol increases number of circulating EPCs. Rats were fed with Cilostazol containing diet (Cilostazol group) or normal diet (Control group) from 2 weeks prior to carotid injury until time of sacrifice. Representative microphotographs of triple staining with DiI-acLDL and FITC conjugated BS-1 lectin and DAPI of circulating EPCs cultured for 4days after isolation from peripherial blood just before and at 2 and 4 weeks after balloon injury. (C) Quantification of circulating EPCs detected by DiI-acLDL/FITC-BS-1 lectin double positive cells in control group (n = 8) and the cilostazol-treated group (n = 7) *, *P<*0.001 vs. Control. (D) Characterization of rat peripheral blood-derived EPCs was performed. After 4 days in culture, cells were stained by immunofluorescence with antibodies against CD14, CD45, CD34, CD31, Flk-1, eNOS and vWF.

To assess the number of circulating EPCs in peripheral blood, EPC culture assay was performed by double staining of cultured EPCs with DiI-acLDL and BS1-lectin. (Figure 3-A and 3-B) The number of circulating EPCs exhibited significant two-fold increase in CLZ group compared to control group (239±25 vs. 113±14/mm^2^, *P<*0.001, n = 8) before surgery, and the significant difference of 2.3-fold increase between CLZ group and control group sustained until 2 weeks after surgery (380±53 vs. 163±7/mm^2^, *P<*0.001, n = 5). At 4 weeks after surgery, the number of circulating EPCs in both groups decreased to similar level (98±16 vs. 70±10, NS, n = 8). (Figure 3-C) Since the number of EPCs assessed by EPC culture assay correlates with those assessed by FACS analysis with markers for Sca-1/Flk-1 in mouse peripheral blood-derived mononuclear cells [7], [8], these findings suggest that CLZ has an effect of EPC mobilization from BM and CLZ further boosts the mobilization in subacute phase following vascular injury.

### Characterization of EPCs

EPCs derived from peripheral blood were cultured in EPC differentiation medium for 4 days and characterized by immunofluorescent staining. Most of the EPCs expressed both several leukocyte antigens (CD14, CD45, CD34) and endothelial antigens (CD31, fetal liver kinase 1 (Flk-1), endothelial nitric oxide synthase (eNOS), von Willebrand factor (vWF)). (Figure 3-D).

### Cilostazol Enhances BM-derived EPC Contribution to Re-endothelialization

**Figure 4:**
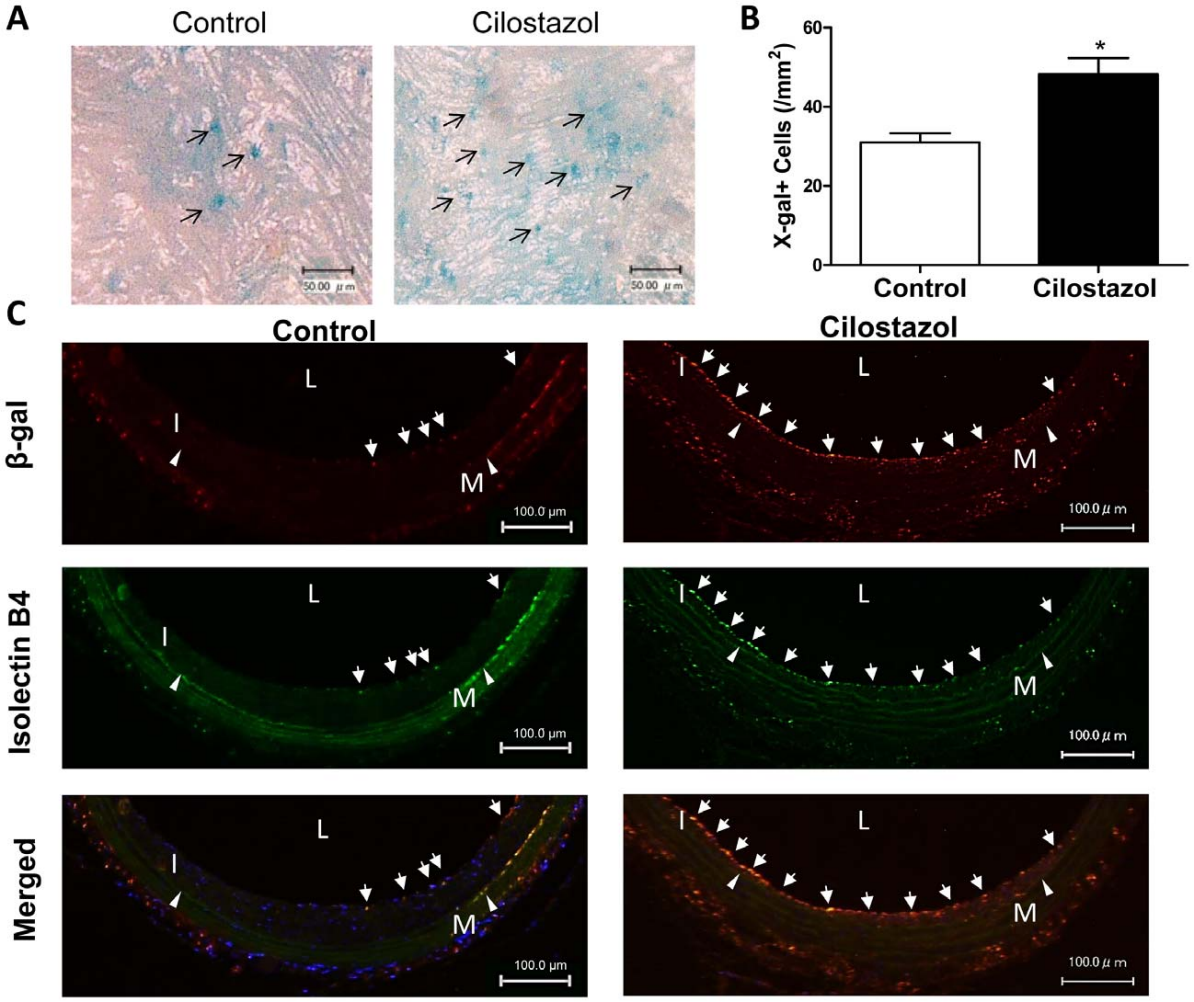
Cilostazol promotes bone marrow-derived EPC recruitment to denuded carotid artery. (A) Nude rats were transplanted with bone marrow of Tie2/lacZ mice and fed with Cilostazol containing diet (Cilostazol group) or normal diet (Control group) from 2 weeks prior to carotid injury until time of sacrifice. Carotid arteries were denuded 6 weeks after bone marrow transplantation and harvested 2 weeks after balloon injury. Representative photomacrographs of luminal surface of X-gal stained injured. (n = 5 in each group) (B) Number of X-gal-positive (blue) cells on luminal surface was counted and averaged. **P<0.01vs. Control. (C) Representative photographs of double immunofluorescence staining for β-galactosidase (β-gal, red) and isolectin B4 (green) with cross sections at 2weeks after carotid injury. IEL, internal elastic lamina (Arrowheads); I, intima; L, lumen; and M, media. Arrows indicate β-gal and isolectin B4-double positive cells.

To assess the contribution of BM–derived EPCs to accelerated re-endothelialization, BM from Tie2/lacZ mice was transplanted to nude rats, and carotid arteries were harvested from Tie2/LacZ BM transplanted nude rats 2 weeks after balloon injury. In this model, BM-derived EPCs originated from donor transgenic mouse are detected by β-galactosidase expression by LacZ gene which is regulated by endothelial specific Tie2 promotor. The β-galactosidase expression was identified by X-gal chemical staining or by immunochemical staining in tissue samples. The number of X-gal-positive cells on luminal surface was significantly greater in CLZ group (n = 5) cells/mm^2^ than that in control group (n = 5) (48±4 vs. 31±2 cells/mm^2^, *P<*0.01). (Figure 4A and 4B) In addition, BM–derived Tie2/LacZ-positive EPCs were further identified by double-fluorescence immunostaining for β-galactosidase and the endothelium-specific marker isolectin B4 with cross sections at 2 weeks after carotid injury. In cross sections of carotid arteries from control group, only a few β-gal-positive and isolectin B4 double-positive cells were observed on the luminal surface. In contrast, numerous double-positive cells were observed on the re-endothelialized luminal surface in carotid arteries from CLZ-treated animals. (Figure 4C) These findings thus suggest that accelerated re-endothelialization achieved with CLZ involves enhanced EPC recruitment to the regenerated neoendothelium.

### Cilostazol Increases EPC Functions In Vitro

**Figure 5:**
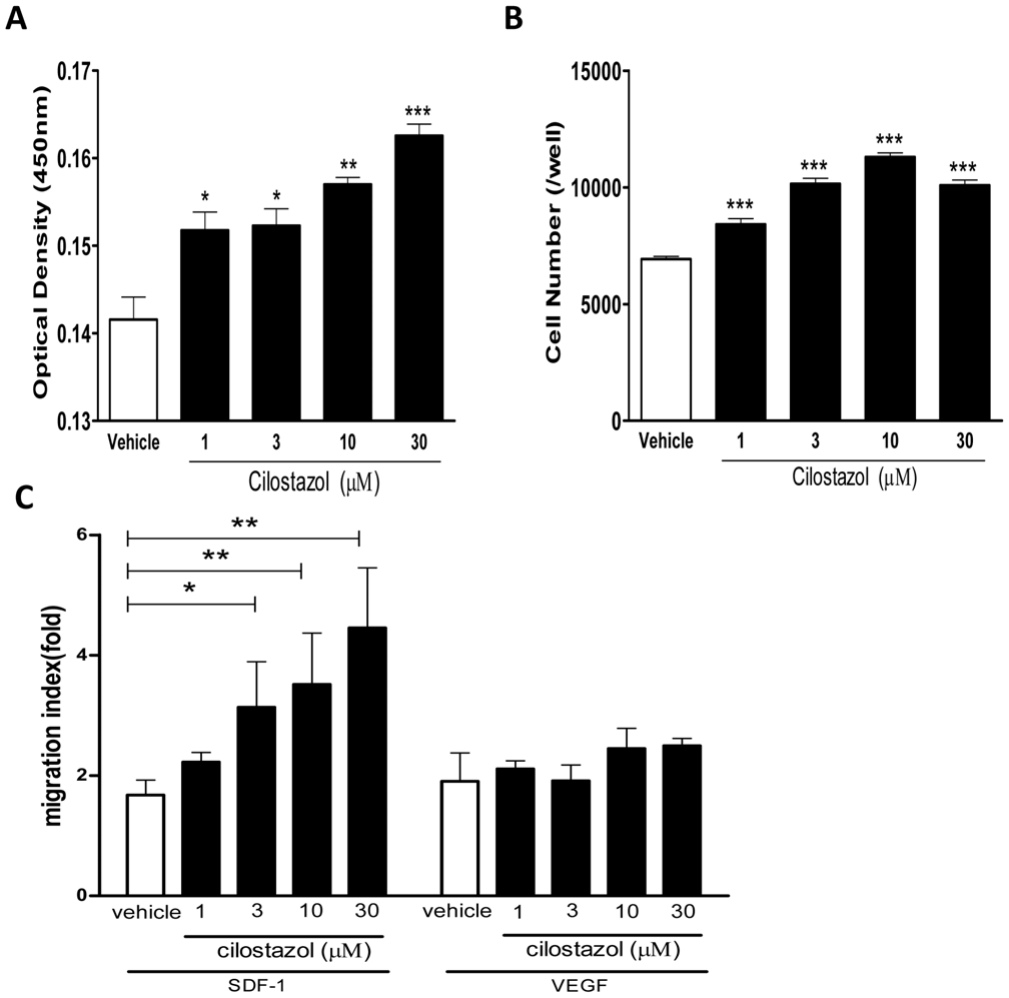
EPC functional assays by Cilostazol treatment. (A) Cilostazol increases EPC proliferation activity. Cultured EPCs were treated with Cilostazol at the indicated concentrations for 3 h and incubated for further 48 h and proliferation activity was examined by colorimetric assay system. *, *P<*0.05; **, *P<*0.01; and ***, *P<*0.001 vs. Vehicle. (B) Cilostazol increases EPC adhesion activity. Cultured EPCs were treated with Cilostazol at the indicated concentrations for 3 h and reseeded on a 96-well plate (5×10^4^cells/well) with Pronectin F and incubated. After one hour in culture, adherent cells were fixed and stained with DAPI. DAPI positive cells were counted in 6 different wells under fluorescent microscope (100X) and averaged. *, *P<*0.001 vs. Vehicle. (C) Cilostazol increases EPC migration activity. Modified Boyden's chamber assay was performed. Cultured EPCs were treated with Cilostazol at the indicated concentrations for 3 h. Cells (1×10^5^cells) were placed in upper chamber and lower chamber was filled with medium containing SDF-1α (100 ng/ml) or VEGF (50 ng/ml) or no chemoattractant (negative control) and incubated for 16 h. Migrated cells were counted following H.E. staining and the migration activity was expressed as a migration index calculated by dividing the number of migrated cells in the presence of SDF-1α or VEGF by the number of migrated cells in the negative controls. *, *P<*0.05 and **, *P<*0.01 vs. Vehicle. All experiments were performed in triplicate and confirmed the reproducibility.

To explore active mechanism of CLZ on rat EPCs, we performed a series of in vitro studies. The proliferation activity of EPCs pre-incubated with CLZ was significantly increased compared with that of vehicle-treated EPCs in a dose dependent manner. (Optical density at 450 nm wavelength: Vehicle 0.143±0.002 vs. 1 µM 0.152±0.002, *P<*0.05; 3 µM 0.152±0.002, *P<*0.05; 10 µM 0.157±0.001, *P<*0.01; 30 µM 0.163±0.001, *P<*0.001). (Figure 5A).

Next, cultured EPCs were incubated with CLZ at the indicated concentrations for 3 hours for assessment of adhesion activity. The adhesion activity of EPCs pre-incubated with CLZ was significantly increased, and CLZ-induced adhesion activity effect was enhanced peaking at a dose of 10 mM. (Adhered cell number: Vehicle 6994±108 vs. 1 µM 8418±241; 3 µM 10156±237; 10 µM 11303±176; 30 µM 9954±231 cells/well, *P<*0.001). (Figure 5B).

The migration activity in response to SDF-1α of EPCs pre-incubated with CLZ was significantly increased compared with vehicle-treated EPCs in a dose dependent manner. (Migration index: Vehicle 1.68±0.25 vs. 1 µM 2.23±0.16, NS; 3 µM 4.24±0.46 *P<*0.05; 10 µM 4.68±0.67, *P<*0.001; 30 µM 4.46±1.00, *P<*0.001) However, there was no promotional effect of CLZ on VEGF-induced EPC migration activity. (Figure 5C) This finding allowed us to focus on the association of CLZ and SDF-1α/CXCR4 signaling pathway rather than VEGF signaling pathway in EPC biological functions.

### Cilostazol Enhances EPC Differentiation and Homing-related Gene Expression

**Figure 6:**
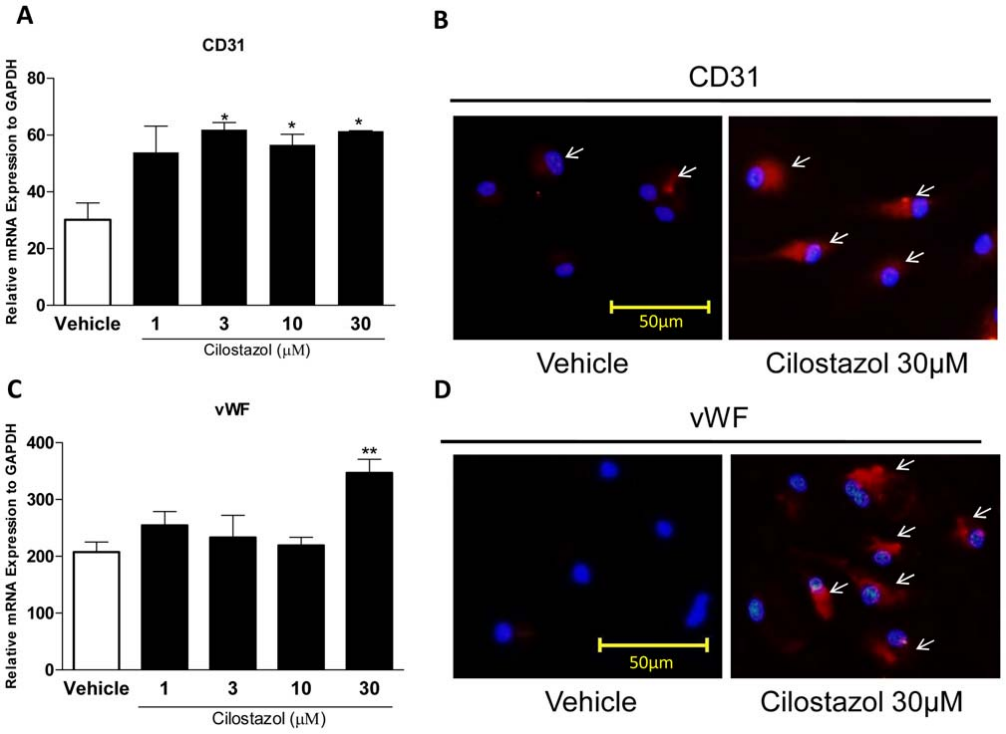
Cilostazol promotes EPC differentiation to endothelial lineage. Cultured EPCs were treated with Cilostazol at the indicated concentrations for 3 h and cultured for an additional 48 hours in EPC differentiation medium. Differentiation activity was examined by real-time RT-PCR analyses for CD31 (A) and vWF (C) mRNA expressions as endothelial markers. *P<0.05 and **P<0.01vs. Vehicle. Representative immunofluorescence photomicrographs for CD31 (B) and vWF (D) in cultured EPCs treated with Cilostazol. Arrows indicate CD31- and vWF-positive cells. All experiments were performed in triplicate and confirmed the reproducibility.

To investigate whether CLZ has an impact on EPC differentiation, the effect of CLZ on mRNA expressions of EC lineage markers CD31 and vWF was examined by quantitative real-time RT-PCR at 2 days of growth after 3-hour CLZ treatment with the indicated concentrations. The mRNA expressions of CD31 and vWF were significantly upregulated in cultured EPCs with CLZ at doses of 3, 10, and 30 µM (Figure 6A) and only 30 µM (Figure 6C), respectively. We next further confirmed the protein expressions of CD31 and vWF in cultured EPCs by immunofluorescent staining. Numerous staining positive cells for CD31 (Figure 6B) and vWF (Figure 6D) were observed at 2 days of growth after 3-hour CLZ (30 µM) treatment, while only a few cells stained positive for CD31and vWF without CLZ. Thus, by evaluating the expression of 2 different EC markers, CLZ directly enhanced EPC differentiation to EC lineage in vitro.

**Figure 7:**
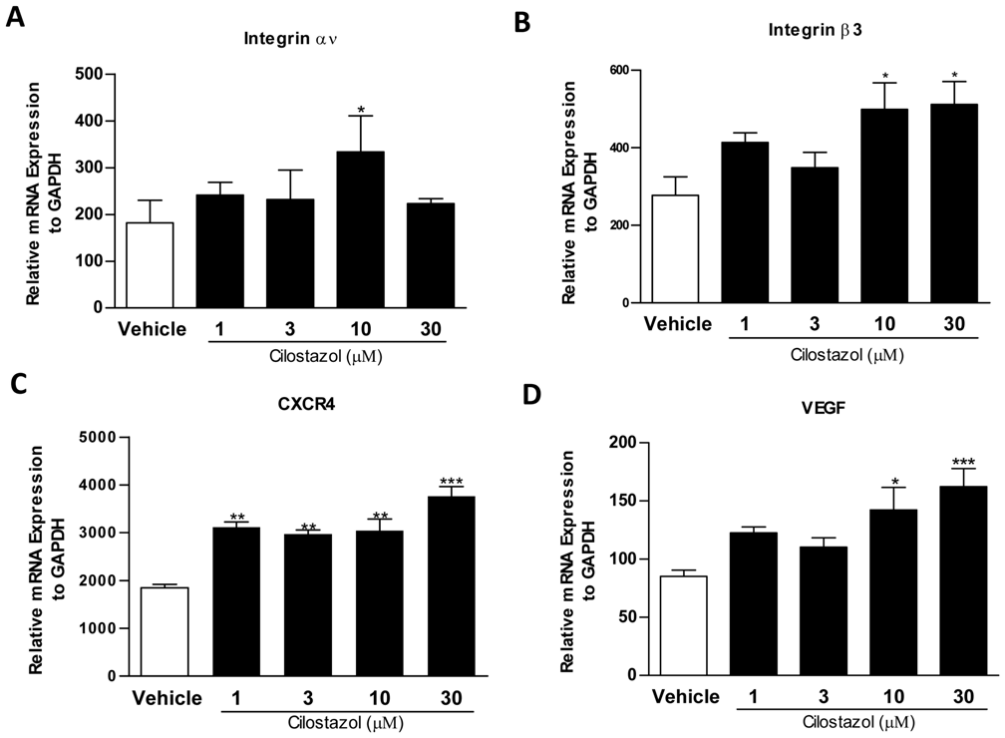
Cilostazol alters gene expression profile in cultured EPCs. Cultured EPCs were treated with Cilostazol at the indicated concentrations for 3 h, and total RNA was extracted. The expressions of adhesion molecule integrin αv(A)/integrin β3 (B), chemokine receptor CXCR4 (C) and growth factor VEGF (D) were examined by quantitative real-time RT-PCR analysis. *, *P<*0.05, **, *P<*0.01 and *** *P<*0.001 vs. Vehicle. All experiments were performed in triplicate and confirmed the reproducibility.

Based on the result of EPC adhesion/proliferation activity increase by CLZ and migration activity increase in response to SDF-1α but not VEGF as described above, the mRNA expression of integrin αv and integrin β3 which are representative adhesion molecules in EPCs, CXCR4 which is a receptor for SDF-1α, and VEGF which is a critical growth factor for EPC differentiation, migration, and proliferation were examined by quantitative real-time RT-PCR. EPCs were grown for 5 days and were then incubated with CLZ for 3 hours at the indicated concentrations. The mRNA expressions of integrin αv (Figure 7A) and integrin β3 (Figure 7B) were significantly upregulated by CLZ treatment at a dose of 10 µM and doses of 10 µM and 30 µM, respectively. CLZ treatment also upregulated CXCR4 mRNA expression (Figure 7C) at any concentrations and did VEGF mRNA expression (Figure 7D) at doses of 10 µM and 30 µM. These findings will be able to explain the reason of EPC functional activation by CLZ treatment.

### Cilostazol Enhances Medial Expression of SDF-1α in Injured Artery

**Figure 8:**
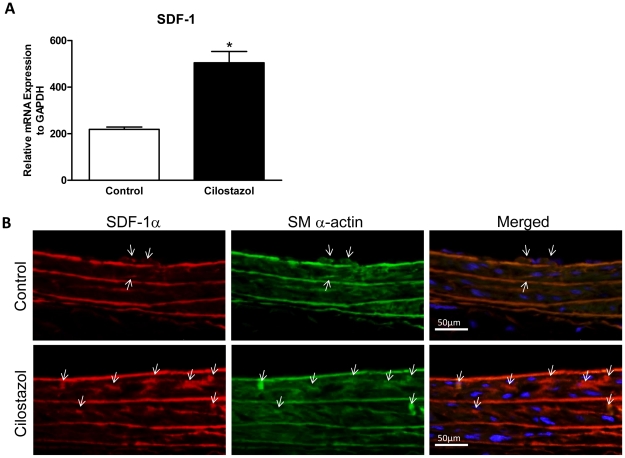
Cilostazol enhances SDF-1 expression in injured carotid artery. Rats were fed with Cilostazol containing diet (Cilostazol group) or normal diet (control group) from 2 weeks prior to carotid injury until time of sacrifice, and inured arteries were examined at 7 days after surgery. (A) The expressions of SDF-1 mRNA were examined by quantitative real-time RT-PCR analysis. *, *P<*0.001 vs. Control. (B) Representative photomicrographs of double-immunofluorescent staining for SDF-1α shown in red and SM α-actin shown in green on histological cross sections in Cilostazol group (n = 4) vs. Control group (n = 3). Arrows indicate double-positive cells for SDF-1α and SM α-actin.

We then examined the expression of SDF-1α in injured artery one week after surgery by both quantitative real-time RT-PCR and immunofluorescent staining. The SDF-1α mRNA expression was significantly upregulated in the CLZ–treated rats compared with that in control rats. (Figure 8A) Next, SDF-1α-positive cells were identified in injured vascular wall by double-fluorescent immunostaining for SDF-1α and smooth muscle (SM) α-actin with cross sections. Only a few/SM α-actin double positive cells were observed in the media in control animals. In contrast, numerous double-positive cells were observed in the media in the CLZ-treated animals (Figure 8B). These data suggest that CLZ enhance the expression of SDF-1α in the injured artery, specifically, in the medial vascular smooth muscle cells (VSMCs). Also, the production of SDF-1α from medial VSMCs might contribute to the recruitment of CXCR4 positive circulating EPCs.

## Discussion

In the present study, we have demonstrated novel biological effects of CLZ on vascular remodeling following arterial injury, specifically, involving BM-derived EPC contribution to re-endothelialization which is a critical response to vascular injury in terms of inhibiting neointima formation. The major findings of this study are: 1) CLZ inhibits neointima formation accelerating re-endothelialization in injured artery, 2) CLZ-inducd accelerated re-endothelialization is mediated by EPC mobilization from BM and circulating EPC recruitment to neoendothelium, 3) CLZ enhances functional properties, adhesion, migration proliferation, and differentiation upregulating adhesion molecule integrin αvβ3, chemokine receptor CXCR4, and growth factor VEGF mRNA in EPCs, and 4) CLZ markedly increase the expression of SDF-1α, which is a ligand for its receptor CXCR4, in medial VSMCs after injury, suggesting that CLZ accelerates re-endothelialization with enhanced EPC recruitment via a SDF-1α/CXCR4 axis in injured arteries.

EPCs were classified into two major cell types according to their time-dependent appearance in culture, so-called early-outgrowth EPCs and late-outgrowth EPCs. Early-outgrowth EPCs (eoEPCs) were obtained by culturing isolated mononuclear cells for 4–7 days and late-outgrowth EPCs (loEPCs) were appeared after 14–21 days in culture demonstrating acetylated LDL uptake and binding to Ulex lectin with expressions of CD31, CD34 (generally at low levels),VE-cadherin, Flk-1 and vWF. Unlike mature endothelial cells, eoEPCs express a monocyte marker CD14 and a pan-leucocyte marker CD45 [28], [29], [30], [31]. A beneficial effect on endothelial repair after injury has been shown by eoEPCs in previous studies [32], [33], [34], and cultured EPCs we used in this study were also characterized as eoEPCs.

As demonstrated in previous studies, EPCs quickly recruit to sites of vascular injury by cytokines and growth factors [35] and stimulate neighbouring EC migration and proliferation by angiogenic growth factor production [36] contributing to endothelial regeneration in injured arteries. In addition, maintenance of normal number and function of circulating EPCs has been reported to be an important novel endogenous vascular repair factor [37], [38], [39]. Therefore, recent studies have proposed that increase of circulating EPC number and activation of EPC function are unique strategies to enhance EPC-mediated re-endothelialization. The evidence of CLZ-induced EPC mobilization and homing to sites of injured artery for re-endothelialization that we have demonstrated in this study may give rise to a novel therapeutic strategy for vascular remodeling following vascular intervention as an EPC mobilizer/activator. Our study has also indicated that CLZ enhanced EPC functional properties of adhesion, proliferation, and migration exhibiting the following possible mechanistic insight in the pathophysiological role of EPCs in re-endothelialization.

Vitronectin, an extracellular matrix protein, has been shown to influence cellular migration and differentiation [40], [41], [42], and Dufourcq et al. showed that VN expression was upreglated in injured rat carotid artery [43]. Our in vitro data of integrin αvβ3 mRNA upregulation by CLZ treatment in EPCs can therefore explain the enhanced EPC adhesion activity against VN and EPC recruitment to injured vascular wall. Moreover, since interaction of integrin αvβ3 and VN is important for cell differentiation [42], upregulation of integrin αvβ3 in EPCs is also helpful for EPC differentiation to EC lineage following the attachment on de-endothelialized vascular wall. On the other hand, for EPC recruitment to injured vascular wall, a certain chemokine produced from injured artery is also crucial as well as adhesion molecule. Indeed, previous mouse studies have shown that SDF-1α protein was expressed in injured carotid arteries with a marked mobilization of circulating Sca-1^+^Lineage^−^ progenitor cells involving EPCs in peripheral blood resulting in cell homing to sites of re-endothelialization, and neutralization of SDF-1α caused delayed re-endothelialization in injured arteries [44], [45], [46], [47]. The receptor for chemokine SDF-1α, CXCR4, in EPCs are essential for the homing [45] and CXCR4-blocked EPCs could not recruit to injured arteries [33], [48] In contrast, overexpression of CXCR4 by gene transfer improves functional properties of human EPCs and enhances re-endothelialization in injured artery [49]. These evidences clearly show that SDF-1α/CXCR4 axis is critical for EPC recruitment to injured vascular wall, and therefore upregulation of both CXCR4 in circulating EPCs and SDF-1α in injured medial VSMCs by CLZ treatment might synergistically promotes EPC-mediated re-endothelialization and CLZ failed to promote VEGF-induced migration activity of EPCs (Figure 5C) in our study. In addition, since SDF-1α is a releasing chemokine, SDF-1α produced from injured medial VSMCs may remotely influence BM and contribute to EPC mobilization from BM into circulation. Indeed, a previous report [48] and our data that timing of EPC mobilization and recruitment to injured arterial wall were synchronized at 2 weeks after arterial injury (Figure 3 and 4) could support the above speculation.

CLZ is a clinically available phosphodiesterase 3 (PDE3) inhibitor, increasing cellar levels of cAMP, with anti-platelet and vasodilatatory properties [13] and is approved in the US for treatment of patient with intermittent claudication symptoms related to peripheral arterial disease [13], [50]. Although distinct mechanism for the favorable effect of CLZ on angiogenesis has not been shown, one very recent study in which CLZ is shown to enhance neovascularization in hippocampus in a mouse model of transient forebrain ischemia via recruitment of BM-derived EPCs [51] suggest a significant contribution of CLZ-induced EPC mobilization/recruitment to angiogenesis in ischemic tissue. For mechanistic insight, as a previous report demonstrated, CLZ increases NO production by the phosphorylation of eNOS with increased cAMP levels and enhances endothelial tube formation in ECs. [52] Since eNOS phosphorylation in BM, in which endothelial/vascular niche for stem/progenitor cells including EPCs is involved, is also crucial for EPC mobilization [4], [53], eNOS phosphorylation could be one of the mechanisms of CLZ-induced EPC mobilization. Furthermore, CLZ might induce eNOS phosphorylation in EPCs themselves as well as in ECs resulting in EPC functional activation as Statins did [54]. CLZ has also been shown to reduce post-procedural in-stent restenosis after arterial stenting [18], [19], [20]. DES has dramatically reduced the rate of restenosis; however, DESs also appears to delay re-endothelialization [1]. This delay results in late angiographic in-stent thrombosis leading to such as an acute coronary syndrome. For instance, Sirolimus, one of the coating materials of DES, accelerates senescence and inhibit proliferation and differentiation, migration of EPCs [55], [56] and EC dysfunction [57], thereby delayed re-endothelialization [58]. In terms of avoiding adverse side effects of DES, CLZ treatment following arterial stenting with DES could be an useful anti-platelet/coagulation therapy for preventing in-stent thrombosis rather than the other anti-platelet agents i.e aspirin, ticlopidine, IIb/IIIa antagonist, and so forth.

In conclusion. the results in the present study provided novel evidences that CLZ inhibited neointima formation following arterial injury accelerating endothelial regeneration with enhancement of BM-derived EPC mobilization, EPC recruitment to sites of injured vessel wall, and EPC function. CLZ could be a promising agent for not only just anti-platelet medicine but also a tool for vascular regenerative medicine.

## Materials and Methods

All procedures and animal care were approved by the Wakayama medical university Institutional Animal Care and Use Committee (Approval Number: 351) and the Ethical Committee in Institute of Biomedical Research and Innovation (IBRI)/RIKEN Center for Developmental Biology (Approval Number: AH21-02), and complied with the Japanese Physiological Society Guidelines for the Care and Use of Laboratory Animals.Cilostazol{6-[4-(1-cyclohexyl-1H-tetrazol-5-yl)butoxy]-3,4-dihydro-2-(1H)-quinolinone} (CLZ) were gifted from Otsuka Pharmaceutical (Tokushima, Japan). The Male Sprague-Dawley (SD) rats (16 to 19 weeks old, 350 to 450 g) were divided into two groups. One group was fed a standard rat diet (control group), and the other group was given a 0.2% CLZ mixed diet (CLZ group) resulting in approximately 60∼80 mg/kg/day of CLZ intake. Previous study showed 50 mg/kg/per day of CLZ intake for 14 days in rat was1.43±0.90 mmol/L of plasma concentration [59],therefore, plasma CLZ level in this study could be equivalent to the dose in human cases [60]. CLZ containing special diet was given 2 weeks before and 2 or 4weeks after carotid injury until they were euthanized. The rats were anesthetized and underwent carotid balloon denudation as described previously [61]. The carotid arteries were examined histologically and blood samples were collected for circulating EPC count before and 2 or 4 weeks after balloon injury. Detailed materials and methods are available in Text S1 and Table S1 and Table S2.

## Supporting Information

Text S1 **Supplementary Materials and Methods.**
(DOCX)

Table S1 **Antibodies used in Immunocytochemical and Immunohistochemical Analyses.**
(DOC)

Table S2 **Primers used in Real-time RT-PCR Analysis.**
(DOC)
