# Correction: Cilostazol Activates Function of Bone Marrow-Derived Endothelial Progenitor Cell for Re-endothelialization in a Carotid Balloon Injury Model

**DOI:** 10.1371/annotation/39d3625c-a28f-4669-9204-4a433d2b4b27

**Published:** 2012-08-10

**Authors:** Rie Kawabe-Yako, Masaaki Ii, Osamu Masuo, Takayuki Asahara, Toru Itakura

The second author's name was spelled incorrectly. The correct name is: Masaaki Ii. The correct citation is: Kawabe-Yako R, Ii M, Masuo O, Asahara T, Itakura T (2011) Cilostazol Activates Function of Bone Marrow-Derived Endothelial Progenitor Cell for Re-endothelialization in a Carotid Balloon Injury Model. PLoS ONE 6(9): e24646. doi:10.1371/journal.pone.0024646 

