# Supplementary material for: Cilostazol Activates Function of Bone Marrow-Derived Endothelial Progenitor Cell for Re-endothelialization in a Carotid Balloon Injury Model
Source: PLoS One. 2011 Sep 12;6(9):e24646. doi: 10.1371/journal.pone.0024646 (PMC3171459; doi:10.1371/journal.pone.0024646)
Supplement: Text S1 — Supplementary Materials and Methods. (DOCX) [file pone.0024646.s001.docx]

**Text S1.Supplementary Materials and Methods.**

**Balloon Injury of Rat Carotid Artery**

SD rats were anesthetized with isoflurane by mask ventilation (Air: 400~500ml /min and Isoflurane: 1.5~2.0%). The right carotid artery was exposed by a midline neck skin incision, and a guidewire (X-pedion-10; eV3. Neurovascular) was inserted from the external carotid artery and advanced retrogradely into the common carotid artery. Then, single-lumen balloon catheter (HyperForm 4×7mm; eV3. Neurovascular) was navigated along the guidewire. After adjusting the proximal end of the balloon to the bifurcation, the balloon was inflated with 0.1 ml saline and maintained for 10 seconds. Balloon inflation was performed six times with rotation through 60°. Balloon injury was completed at 7 mm proximal to the carotid bifurcation followed by ligation of the external carotid artery. Sham operation was performed in a similar surgical procedure without balloon injury.

**Histological Analysis**

Re-endothelialization was evaluated as described previously, [[1](#_ENREF_1),[2](#_ENREF_2)] assessments were performed in CLZ group and control group. Briefly, Evans blue dye (Sigma-Aldrich Co., St. Louis, MO, USA) diluted with saline (5%) was injected into the heart, the heart was perfused with saline to wash out the whole blood and excess dye, then the stain was fixed via perfusion of 4% paraformaldehyde (PFA) in phosphate-buffered saline (PBS). Ten minutes after PFA perfusion, rats were sacrificed, and carotid arteries were harvested for *en face* histological assessments; regions of damaged endothelium incorporate the stain and appear blue whereas re-endothelialized tissue is resistant to the dye. The areas stained blue and the unstained areas were outlined and measured with Image J^TM^ software. For determination of intima/media (I/M) ratio, serial cross sections of paraffin-embedded specimens were stained with hematoxylin and eosin. The intimal area and the medial area were measured with Image J^TM^ software in each section and I/M ratio was calculated and averaged.

**Rat BM Transplantation Model and Fluorescent Immunohistochemistry of Carotid Arteries**

Rat BM transplantation was performed as described previously.[[3](#_ENREF_3),[4](#_ENREF_4),[5](#_ENREF_5)] Briefly, lethally irradiated nude rats (F344/NJcl-*rnu*/*rnu* 220~250g weight; CLEA Japan, Tokyo) received BM cells from transgenic Tie2/lacZ mice, which constitutively express β-galactosidase encoded by lacZ under the transcriptional regulation of an endothelium-specific promoter, Tie 2 (Jackson Laboratories). Four weeks after BM transplantation, nude rats were divided into two groups of CLZ group (CLZ mixed diet) and control group (normal diet). Carotid arteries were denuded 6 weeks after BM transplantation with a wire technique. Sham operation of the contra-lateral side was performed in the same manner. Nude rats were euthanized and the carotid arteries were harvested at 2 weeks after carotid denudation.

X-gal staining was performed on whole-mount en face injured carotid arteries to visualize and quantify BM–derived Tie2/lacZ-positive cells per square millimeter of surface area as described previously.[[6](#_ENREF_6)] Double immunofluorescent staining was also performed on cross sections to identify BM–derived Tie2/lacZ-positive endothelial lineage cells with an antibody against β-galactosidase and isolectin B4. Rabbit polyclonal anti-mouse β-galactosidase antibody (Cortex,San Leandro,California,USA) was used at 1:800 dilution at 4°C overnight, and an Alexa Fluor594-conjugated Goat anti-rabbit IgG polyclonal was used as a secondary antibody at 1:1000 dilution at room temperature for 30minites. Endothelium-specific isolectin B4 conjugated with fluorescein isothiocyanate (FITC; Vector) was used at 1:200 dilution at 4°C overnight. Normal rabbit IgG served as a negative control. The resulting fluorescent signal was detected by a fluorescent microscopy.

**Rat EPC Culture Assay**

Rat mononuclear cells (MNCs) were initially isolated from 500µl of peripheral blood at before and 2 or 4 weeks after balloon denudation by density gradient centrifugation with Histopaque-1083 (Sigma-Aldrich, St.Louis, USA) and incubated in 10% fetal bovine serum(FBS)/ EGM-2-MV BulletKit (Clonetics, San Diego, CA) medium on rat vitronectin (Sigma-Aldrich) /gelatin coated 4-well chamber slides. After 4days in culture, to detect the uptake of 1,1’-dioctadecy1-3,3,3’,3’-tetramethylindocarbocyanine-labeled acetylated low-density lipoprotein (DiI-acLDL; Biomedical Technologies, Stoughton, USA), cells were incubated with DiI-acLDL (10µg/ml) at 37°C for 1 hour. Cells were then fixed with 0.5% PFA for 10 minutes, and incubated with FITC-conjugated Bandeiraea simplicifolia agglutinin( BS-1 lectin, 20µg/ml; Sigma-Aldrich) at 4°C 4hours. After the staining, samples were examined with an inverted fluorescent microscope (BioZero, KEYENCE, Tokyo, Japan). Dual-stained cells for both FITC-BS1 lectin and DiI-acLDL were considered as EPCs, and the number of EPCs per square millimeter was counted in a blinded fashion.

**Characterization of circulating EPCs**

The cultured circulating EPCs were examined by immunofluorescentcytochemistry with antibodies against CD14, CD45, CD34, CD31, Flk-1, eNOS and vWF. The cells were also stained with 4',6-diamidino-2-phenylindole (DAPI: Wako Pure Chemical Industries Ltd., Osaka, Japan) for nuclear counter staining. Negative controls were set by normal IgGs instead of using primary antibodies in PBS. The information of antibodies and staining conditions used in this immunofluorescent staining are shown in **Table S1**. The fluorescent signals were detected under a fluorescence microscopy.

**Rat BM-derived EPC Culture**

Rat EPCs were isolated by culture method as described previously.[[7](#_ENREF_7)] BM-derived mononuclear cells isolated from femoral bones were cultured on dishes (1×10^6^/cm^2^) in 10%FBS/DMEM (Invitrogen) medium. After 2days in culture, nonadherent cells were reseeded in 10%FBS/EGM-2-MV BulletKit (Clonetics). After 2 days in further culture, attached cells were used as EPC-rich cell population for in vitro study.

**Function Assays for EPC Adhesion, Migration and Proliferation Activities**

EPC proliferation activity was evaluated using a Cell-counting kit 8 (Doujindo Molecular Technologies, Inc., Kumamoto, Japan). EPCs were seeded on ProNectin F^TM^ coated 96-well culture plates (1×10^4^cells/well) and incubated in 10%FBS/EGM-2-MV BulletKit for 24 hours. EPCs were then incubated for 16 hours in 0.5%BSA/EBM-2 and treated with CLZ at 1, 3, 10 and 30μM or vehicle for 3 hours and incubated in 10%FBS/EGM-2-MV BulletKit for further 48 hours. Cell-counting kit 8 solution was then added to each well and the cells were cultured for an additional 4 hours. The light absorbance at 450nm was measured using an enzyme-linked immunosorbent assay (ELISA) plate reader and averaged.

EPC adhesion to extracellular matrices was evaluated as described previously.[[5](#_ENREF_5)] EPCs were rendered quiescent by incubation for 16h in 0.5% BSA/EBM-2 and were stimulated with CLZ at 1, 3, 10 and 30μM or vehicle for 3 hours and reseeded on 96-well culture plate (5×10^4^cells) pre-coated with ProNectin F^TM^ (Sanyo Chemical, Kyoto, Japan) and incubated for 1 hour at 37°C and 5%CO_2_. After washing 3 times with PBS, attached cells were stained by DAPI. DAPI positive cells were counted. Adhesion activity was evaluated as the mean number of attached cells in one high power fields (×10) from 6wells.

EPC migrations were evaluated using a modified Boyden’s chamber assay as described previously.[[8](#_ENREF_8),[9](#_ENREF_9)] 24-well transwell culture plates with 5 μm-pore size-polycarbonate filter (Kurabo Industries, Osaka, Japan) were used. Briefly, EPCs were incubated for 16 hours in EBM-2/0.5%BSA and treated with several concentrations of CLZ for 3 hours. Cell suspensions (1×10^5^ cells/well) were placed in the upper chamber and the lower chamber was filled with 0.5%BSA/EBM-2 medium only as negative control or containing rat stromal cell-derived factor-1α (SDF-1α) (100 ng/mL: R&D Systems, Minneapolis) or rat vascular endothelial growth factor (VEGF)(50 ng/mL: R&D Systems, Minneapolis). The chamber was incubated for 16 hours at 37°C and 5% CO2. Migrated cells were stained with H&E and the migration activity was expressed as a migration index calculated by dividing the number of migrated cells in the presence of SDF-1α or VEGF by the number of migrated cells in the negative controls.

**Differentiation assay with BM-derived cultured EPCs**

To investigate whether CLZ has an impact on EPC differentiation, the effect of CLZ on mRNA expressions of EC-specific markers, CD31 and vWF, was examined. The expressions of CD31 and vWF mRNA were analyzed by quantitative real-time RT-PCR at 2 days of growth after 3-hour CLZ treatment at the indicated concentrations. In addition, fluorescence immunostaining was performed with antibodies against CD31 and vWF. The cells were also stained with 4', 6-diamidino-2-phenylindole (DAPI: Wako Pure Chemical Industries Ltd., Osaka, Japan) for nuclear counter staining. Normal rabbit IgG was served as a negative control. The information of antibodies and staining conditions used in this immunofluorescent staining are shown in **Table S1**. The fluorescent signals were detected under a fluorescence microscopy.

**Quantitative Real-time RT–PCR Analysis in EPCs**

The mRNA expressions of an adhesion molecule, integrin subunits αv/β3; endothelial lineage markers, CD31 and vWF; a growth factor, VEGF; and a receptor for SDF-1α, CXCR4 were examined by real-time reverse transcription–polymerase chain reaction (RT-PCR) in BM-derived EPCs. Rat BM-derived EPCs were incubated for 16 hours in 0.5%BSA/EBM-2 and treated with several concentrations of CLZ for 3 hours. Total RNA was then extracted by using an RNeasy Mini Kit (QIAGEN Science, Hilden, Germany.) from EPCs according to the manufacturer’s instructions. Reverse transcription were performed using PrimeScript^TM^ II 1st strand cDNA Synthesis Kit (Takara Biochemicals, Kyoto, Japan.) according to the manufacturer’s instructions. For quantitative RT-PCR, the converted cDNA samples (2 μL) were amplified in triplicate by real-time PCR machine (ABI Prism7000, Applied Biosystems, Foster City, CA, USA) in a final volume of 10 μL using SYBR Green Master Mix reagent (Applied Biosystems, Foster City, CA, USA) with gene-specific primers. (**Table S2**) Melting curve analysis was performed with Dissociation Curves software (Applied Biosystems) and the mean cycle threshold (Ct) values were used to calculate gene expressions with normalization to rat GAPDH (rGAPDH).

**Quantitative Real-Time RT-PCR Analysis in Injured Carotid Artery**

Expressions of SDF-1 mRNA in denuded carotid arteries were evaluated by quantitative real-time RT-PCR. Total RNA was extracted from the injured arteries in control group and CLZ group with Trizol (Invitrogen) according to the manufacturer’s instructions 7 days after surgery in each group (*n* = 3 per group). After treatment with DNase I (RNase-free; Takara Biochemicals), cDNA were synthesized from 100ng of total RNA using Superscript^TM^ VILO™ cDNA synthesis kit (Invitrogen) according to the manufacturer's instructions. The synthesized cDNA was amplified by PCR with TaqMan^®^ PreAmp Master Mix (Applied Biosystems). Preamplification reaction volumes were consisted of 2.5μL cDNA template with 4.25 μL of Taq-Man^TM^ PreAmp Master Mix (Applied Biosystems) and 2.5μL each of forward and reverse primers. (**Table S2**) The amplification conditions followed several steps; 10 min at 95 °C followed by 15 cycles of denaturing (95 °C, 10sec), annealing and extension (60 °C, 4min). Real-time PCR was performed using TaqMan^TM^ Gene Expression Master Mix (Applied Biosystems) on an ABI Prism 7000 (Applied Biosystems) according to the manufacturer’s instructions. The relative quantities of mRNA expression of the target gene were determined using comparative Ct method and were normalized against GAPDH.

**Fluorescence Immunohistochemistry of Injured Artery**

The carotid arteries were denuded with balloon catheter in CLZ-treated rats and excised one week after surgery. Serial cross sections of paraffin-embedded specimens were analyzed with fluorescent double-immunostaining with an antibody against SDF-1α and smooth muscle (SM) α-actin. The sections were also stained with DAPI (Wako Pure Chemical Industries Ltd., Osaka, Japan) for nuclear counter staining. Normal rabbit IgG was served as a negative control. The detailed information of antibodies and staining conditions used in this immunofluorescent staining are shown in **Table S1**. The fluorescent signals were detected under a fluorescence microscopy.

**Statistical Analysis**

All values were expressed as mean ± SEM. Statistical analyses were performed with commercially available software (GraphPad Prism^TM^, MDF software, Inc.). Comparisons between multiple groups and two groups were tested for significance via analysis of variance (ANOVA) followed by post-hoc testing with the Tukey procedure and nonparametric Mann-Whitney U test, respectively. A *P* value less than 0.05 was considered significant.

**References**

1. Asahara T, Bauters C, Pastore C, Kearney M, Rossow S, et al. (1995) Local delivery of vascular endothelial growth factor accelerates reendothelialization and attenuates intimal hyperplasia in balloon-injured rat carotid artery. Circulation 91: 2793-2801.

2. Goukassian DA, Kishore R, Krasinski K, Dolan C, Luedemann C, et al. (2003) Engineering the response to vascular injury: divergent effects of deregulated E2F1 expression on vascular smooth muscle cells and endothelial cells result in endothelial recovery and inhibition of neointimal growth. Circ Res 93: 162-169.

3. Asahara T, Takahashi T, Masuda H, Kalka C, Chen D, et al. (1999) VEGF contributes to postnatal neovascularization by mobilizing bone marrow-derived endothelial progenitor cells. EMBO J 18: 3964-3972.

4. Takahashi T, Kalka C, Masuda H, Chen D, Silver M, et al. (1999) Ischemia- and cytokine-induced mobilization of bone marrow-derived endothelial progenitor cells for neovascularization. Nat Med 5: 434-438.

5. Walter DH, Rittig K, Bahlmann FH, Kirchmair R, Silver M, et al. (2002) Statin therapy accelerates reendothelialization: a novel effect involving mobilization and incorporation of bone marrow-derived endothelial progenitor cells. Circulation 105: 3017-3024.

6. Takahashi M, Hakamata Y, Takeuchi K, Kobayashi E (2003) Effects of different fixatives on beta-galactosidase activity. J Histochem Cytochem 51: 553-554.

7. Kahler CM, Wechselberger J, Hilbe W, Gschwendtner A, Colleselli D, et al. (2007) Peripheral infusion of rat bone marrow derived endothelial progenitor cells leads to homing in acute lung injury. Respir Res 8: 50.

8. Ii M, Takenaka H, Asai J, Ibusuki K, Mizukami Y, et al. (2006) Endothelial progenitor thrombospondin-1 mediates diabetes-induced delay in reendothelialization following arterial injury. Circ Res 98: 697-704.

9. Yu Y, Fukuda N, Yao EH, Matsumoto T, Kobayashi N, et al. (2008) Effects of an ARB on endothelial progenitor cell function and cardiovascular oxidation in hypertension. Am J Hypertens 21: 72-77.
