# Supplementary material for: Cilostazol Activates Function of Bone Marrow-Derived Endothelial Progenitor Cell for Re-endothelialization in a Carotid Balloon Injury Model
Source: PLoS One. 2011 Sep 12;6(9):e24646. doi: 10.1371/journal.pone.0024646 (PMC3171459; doi:10.1371/journal.pone.0024646)
Supplement: Table S1 — Antibodies used in Immunocytochemical and Immunohistochemical Analyses. (DOC) [file pone.0024646.s002.doc]

**Table S1. Antibodies used in Immunocytochemical and Immunohistochemical Analyses**

| IgG Type | Antigen | Developer | Incubation Time (h) | Incubation Temperature | Dilution Ratio |
| --- | --- | --- | --- | --- | --- |
| Goat | CD14 | (Santa Cruz Biotechnology,Inc) | 1 | RT | 1:50 |
| Mouse | CD45 | (Santa Cruz Biotechnology,Inc) | 1 | RT | 1:50 |
| Mouse | CD31 | (BD Pharmingen™) | 1 | RT | 1:20 |
| Rabbit | CD34 | (Santa Cruz Biotechnology,Inc) | 1 | RT | 1:50 |
| Rabbit | Flk-1 | (Sigma) | 1 | RT | 1:100 |
| Rabbit | eNOS | (Sigma) | 1 | RT | 1:100 |
| Rabbit | vWF | (CHEMICON International, Inc.) | 1 | RT | 1:100 |
| FITC | SM α-actin | (Sigma) | 1 | RT | 1:200 |
| Rabbit | SDF-1α | (Strath) | 1 | RT | 1:200 |
| Alexa Fluor 594or488 | Mouse IgG1- | Invitrogen | 0.5 | RT | 1:1000 |
| Alexa Fluor 594or488 | Rabbit IgG | Invitrogen | 0.5 | RT | 1:1000 |
| Alexa Fluor 594or488 | Goat IgG | Invitrogen | 0.5 | RT | 1:1000 |

CD31 (PECAM-1) : platelet endothelial cell adhesion molecule 1, Flk-1 : fetal liver kinase 1,eNOS : endothelial nitric oxide synthase, vWF : Von Willebrand factor, SM α-actin : α-smooth muscle actin, SDF-1α: stromal cell-derived factor 1α, RT : room temperature
