# Supplementary material for: Cilostazol Activates Function of Bone Marrow-Derived Endothelial Progenitor Cell for Re-endothelialization in a Carotid Balloon Injury Model
Source: PLoS One. 2011 Sep 12;6(9):e24646. doi: 10.1371/journal.pone.0024646 (PMC3171459; doi:10.1371/journal.pone.0024646)
Supplement: Table S2 — Primers used in Real-time RT-PCR Analysis. (DOC) [file pone.0024646.s003.doc]

**Table S2. Primers used in Real-time RT-PCR Analysis**

| Name | Gene Bank Accession# | Primer Sequence | Probe  (5’-FAM, 3’-BHQ) | Position | Length (bp) |
| --- | --- | --- | --- | --- | --- |
| GAPDH | NM_017008.3 | F:GGCACAGTCAAGGCTGAGAATG | - | 242-384 | 143 |
|  |  | R:ATGGTGGTGAAGACGCCAGTA | - |  |  |
| PECAM1 (CD31) | NM_031591.1 | F:TTGGCACCATGAACAAACTAGCA | - | 1830-1921 | 92 |
|  |  | R:CGCTTCGGAGACTGGTCACA | - |  |  |
| vWF | NM_001013938.1 | F:CCTACACTTTGTGGATGTGGATGAC | - | 614 -734 | 121 |
|  |  | R:GCGGAAGCCATTGGACAGA | - |  |  |
| Integrin αv | NM_001106549.1 | F:GAACCCGATGAAGGCTGGAA | - | 2302-2443 | 142 |
|  |  | R:AGACACGACTGGGCTGACATTG | - |  |  |
| Integrin β3 | NM_153720.1 | F:TTCAATGCCACCTGCCTCAA | - | 1194-1278 | 85 |
|  |  | R:TGAAGCTCACCGTGTCTCCAA | - |  |  |
| VEGF | NM_001110333.1 | F:GTCCTCACTTGGATCCCGACA | - | 2087-2185 | 99 |
|  |  | R:CCTGGCAGGCAAACAGACTTC | - |  |  |
| CXCR4 | NM_022205.3 | F:GACGCCATGGCTGACTGGTA | - | 355-471 | 117 |
|  |  | R:GTAGCGGTCCAGGCTGATGAA | - |  |  |
| GAPDH | NM_017008.3 | F:TGGGTGTGAACCACGAGAAA | TGACAACTCCCTCAAGATTGTCAGCAA |  |  |
|  |  | R:GCAGTTGGTGGTGCAGGAT |  |  |  |
| CXCL12 (SDF-1) | NM_022177.3 | F:CTTGCCGTGAAGCCACAGT | ATTCTGGGTTCCAATCAGAAATGGGAAC |  |  |
|  |  | R:TCTGTGATCATTAGGACGTATCCAA |  |  |  |

GAPDH: glyceraldehyde-3-phosphate dehydrogenase, PECAM-1: platelet endothelial cell adhesion molecule 1, vWF: Von Willebrand factor, VEGF: vascular endothelial growth factor, CXCR4: C-X-C chemokine receptor type 4, CXCL12 : chemokine (C-X-C motif) ligand 12
